# Supplementary material for: Codon bias imposes a targetable limitation on KRAS-driven therapeutic resistance
Source: Nat Commun. 2017 Jun 8;8:15617. doi: 10.1038/ncomms15617 (PMC5472712; doi:10.1038/ncomms15617)

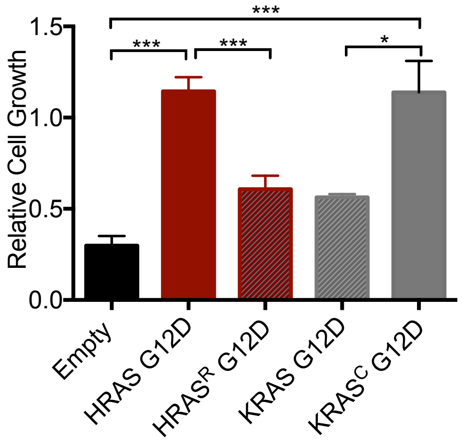

Supplementary Figure 1. Codon bias underlies the differential resistance conferred by oncogenic *HRAS* versus *KRAS*. Relative cell growth quantification for a soft agar growth assay in UACC-62 cells stably expressing the indicated transgenes (or empty vector control) and treated with the BRAF inhibitor PLX4720. Error bars show average ± SEM (n = 3 technical replicates). * signifies $p \leq 0.05$; ** signifies $p \leq 0.01$; *** signifies $p \leq 0.001$.

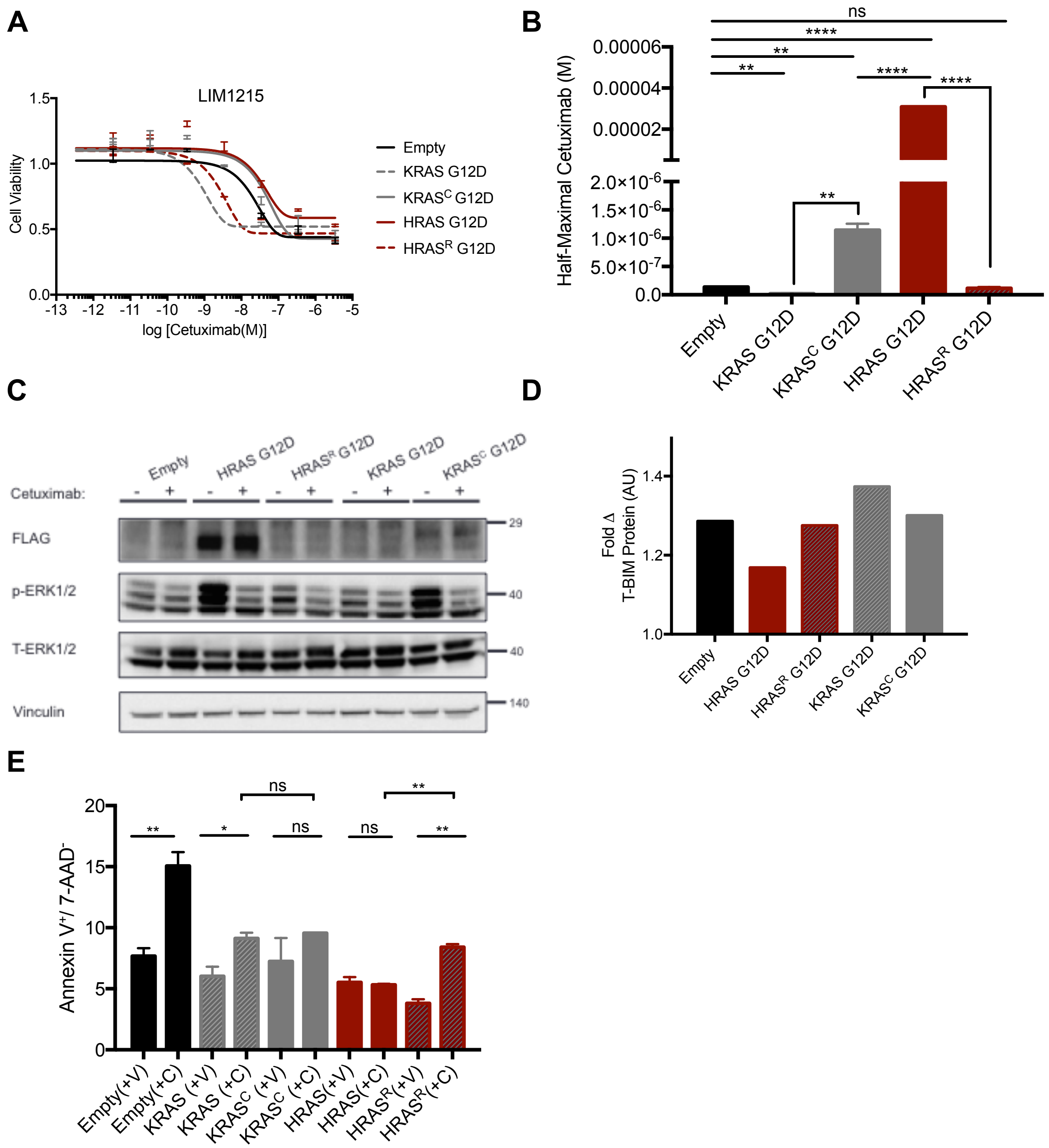

Supplementary Figure 2. Rare codons limit *KRAS*-mediated resistance to cetuximab in colorectal cancer. **(a)** Nonlinear regression growth curves depicting cell viability as detected by Cell Titer Glo of cells transduced with indicated constructs and treated with an eight-log dilution series of cetuximab (LIM1215 cells). **(b)** Derived half-maximal growth inhibition values of curves in panel (a). **(c)** Immunoblotting of indicated constructs for signaling of P/T- ERK1/2. All *HRAS* and *KRAS* constructs are FLAG tagged, and vinculin is a loading control. **(d)** Fold change in extra long BIM (EL-BIM) protein levels from the immunoblots in panel (c) comparing cetuximab and vehicle treated cells. **(e)** Apoptosis quantification (Annexin V^+ / 7-AAD$^-$) of indicated constructs in the presence or absence of cetuximab. In all cases, data are average ± SEM (n = 3 technical replicates per condition). * signifies $p \leq 0.05$; ** signifies $p \leq 0.01$; *** signifies $p \leq 0.001$; **** signifies $p \leq 0.0001$.

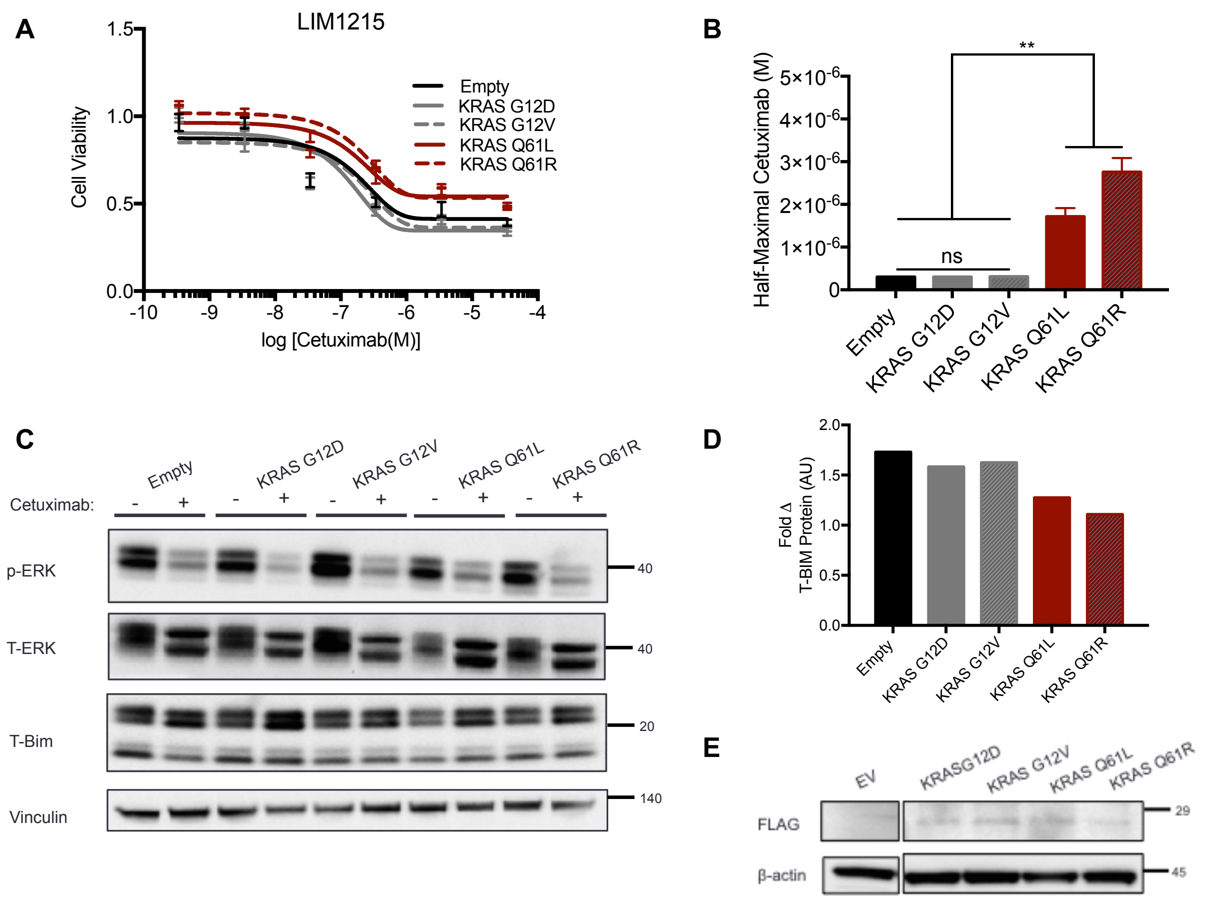

Supplementary Figure 3. Model of clinical *KRAS*-mediated resistance to cetuximab reveals selection of more potent *KRAS* mutations. (**a**) Nonlinear regression growth curves depicting cell viability as detected by CellTiterGlo (CTG) of LIM1215 cells transduced with a vector encoding no transgene (empty) or the indicated RAS transgenes in the presence of cetuximab. (**b**) Derived half-maximal growth inhibition values of curves in panel (a). (**c**) Immunoblotting of indicated constructs for signaling for phospho- and total-ERK1/2 and T-BIM in LIM1215 cells. (**d**) Fold change in total BIM (T-BIM) protein levels from the immunoblots in panel (c) comparing cetuximab and vehicle treated cells. (**e**) Immunoblotting of indicated constructs for FLAG-tagged KRAS. In all cases, data are average ± SEM (n = 3 technical replicates per condition). Images are cropped for clarity. Error bars show data ± SEM. ** signifies $p \leq 0.01$.

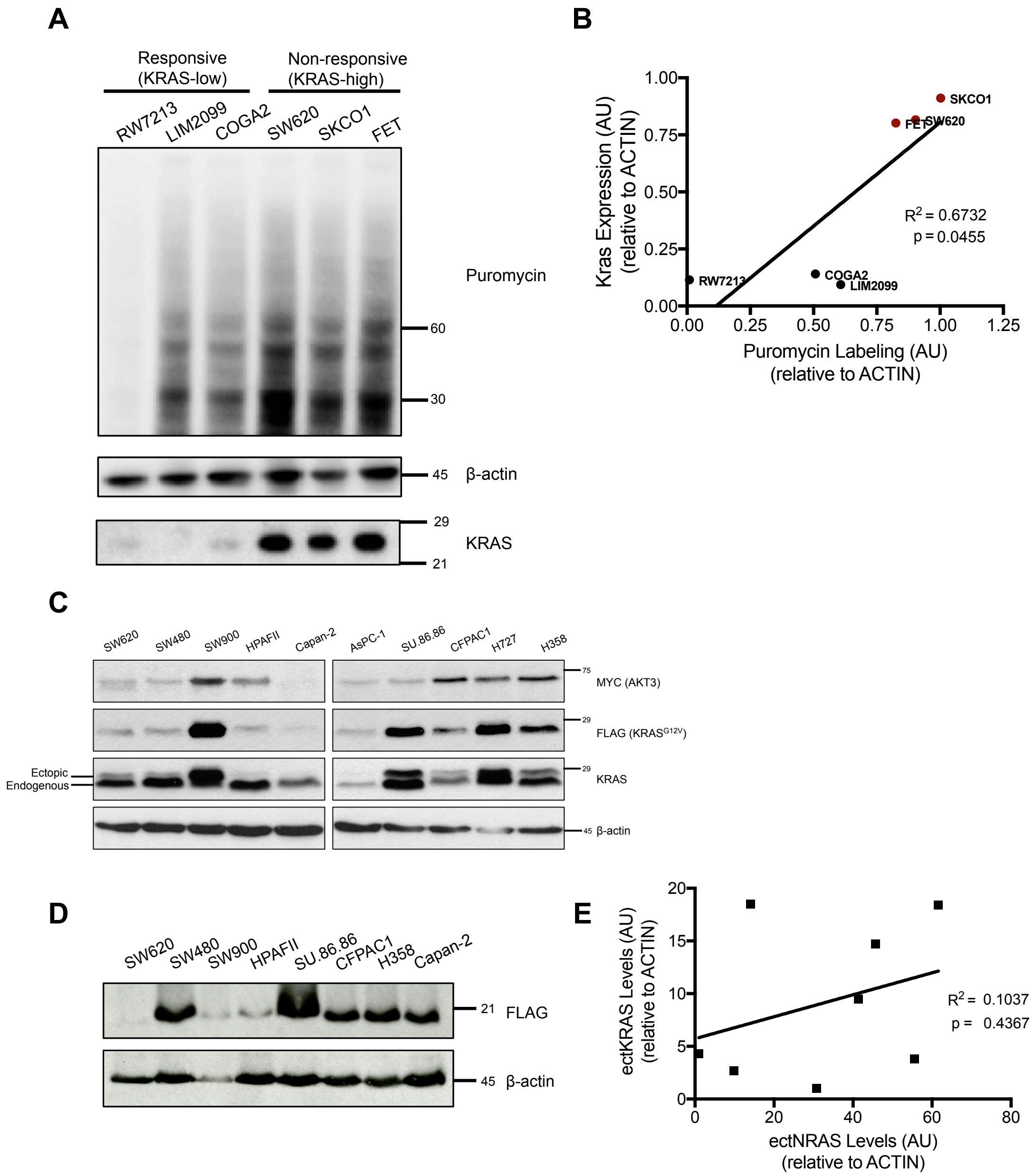

Supplementary Figure 4. Expression levels of mutant *KRAS* correlate with intrinsic resistance to cetuximab and are mediated by translational upregulation. (**a**) Immunoblotting of puromycin labeling following treatment with puromycin for 15 minutes in representative responsive and non-responsive *KRAS* mutant cell lines presented in Figure 5B. (**b**) Linear correlation plot of puromycin labeled protein levels and endogenous KRAS expression of *KRAS* mutant cell lines in (a). (**c**) Immunblotting for endogenous WT KRAS, exogenous FLAG-tagged KRAS^{G12V}, and exogenous MYC-tagged AKT3 in a panel of *KRAS* mutant cell lines. (**d**) Immunoblot analysis for exogenous FLAG-tagged *NRAS* in a panel of *KRAS* mutant cell lines. (**e**) Linear correlation plot of ectopic FLAG-tagged NRAS protein levels and ectopic FLAG- KRAS^{G12V} protein levels in cell lines presented in supplementary Figure 5C. Experiments were completed in duplicate. Images are cropped for clarity.

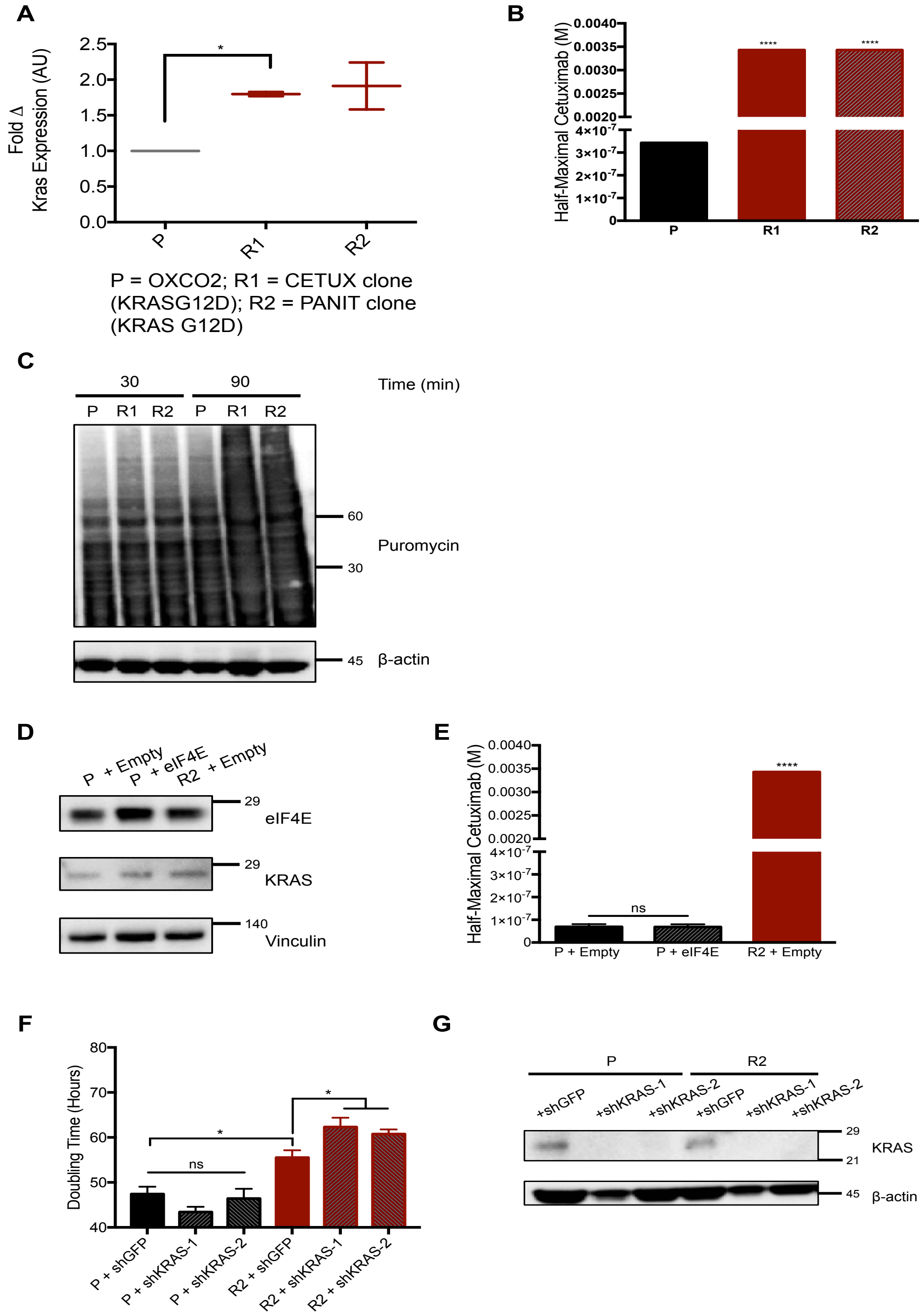

Supplementary Figure 5. Translation-dependent state of *KRAS*^{G12} mutant cells enables selective targeting of anti-EGFR refractory colorectal cancer. (**a**) Fold change in KRAS protein levels, normalized to loading control, between resistant-derivatives (R1 and R2) and parental cells (P) in the OXCO2 cell line. (**b**) Derived half-maximal growth inhibition values of parental (P) and matched resistant-derivatives (R1 and R2) in panel (a). (**c**) Immunoblotting of puromycin labeling at 30 and 90 minutes in parental (P) and derived *KRAS* mutant, cetuximab-resistant (R1 and R2) clones in the LIM1215 cell line. (**d**) Immunblot analysis for endogenous *KRAS* and exogenous eIF4E in a parental (P) and the cetuximab-resistant R2 clone. (**e**) Derived half-maximal growth inhibition values to cetuximab for LIM1215 derivatives presented in panel (d). (**f**) Doubling time, in hours, of various parental (P) and cetuximab-resistant R2 clone derivatives following lentiviral transduction with shGFP or one of two shKRAS hairpins. (**g**) Immunoblotting for endogenous KRAS in parental (P) and derived cetuximab-resistant R2 clone transfected derivatives presented in panel (f). Error bars show data ± SEM (n = 3 technical replicates per condition). * signifies $p \leq 0.05$; **** signifies $p \leq 0.0001$.

Supplementary Figure 6: Uncropped Western Blot Images

Figure 1E

HRAS

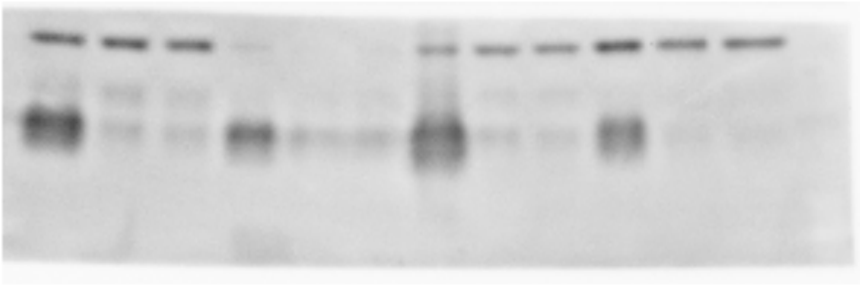

KRAS

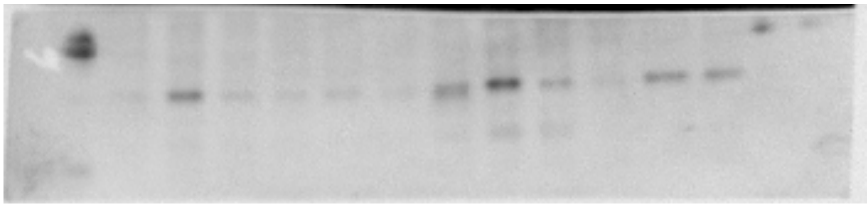

B-actin

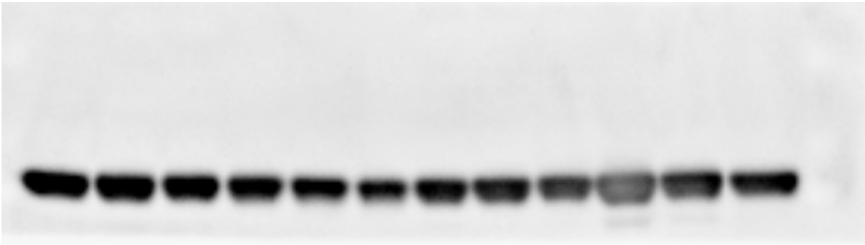

Figure 2:

FLAG B-actin

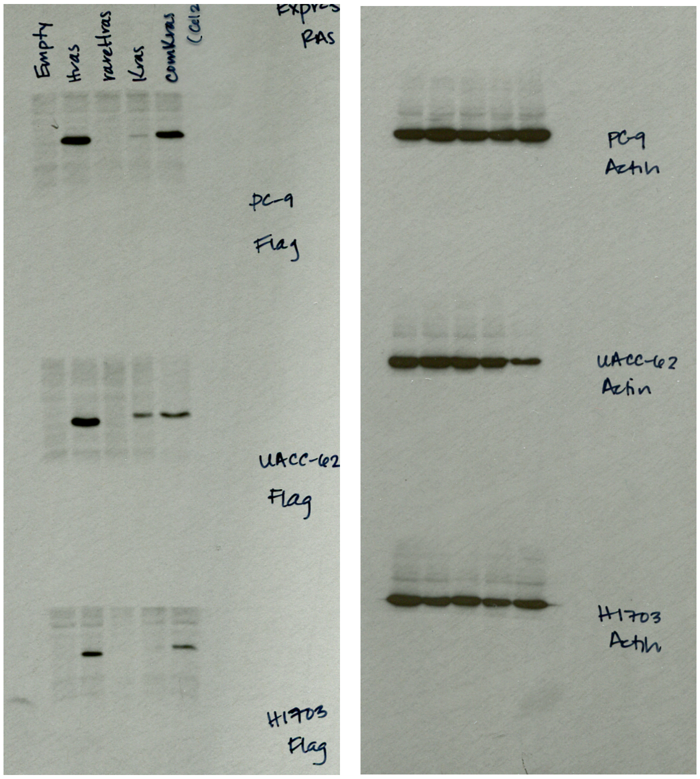

Figure 3:

3A:

HA

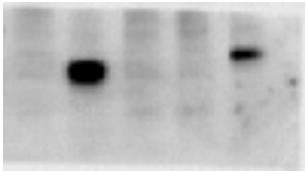

Vinculin

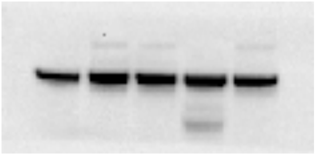

3F:

pERK1/2

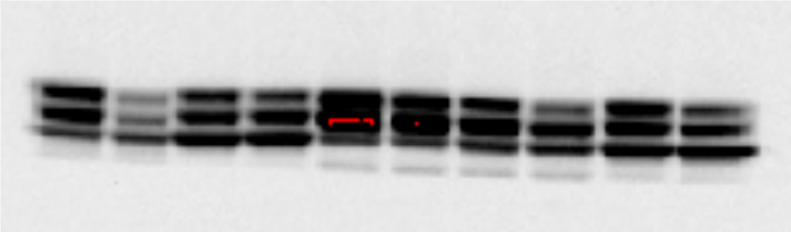

T-ERK1/2

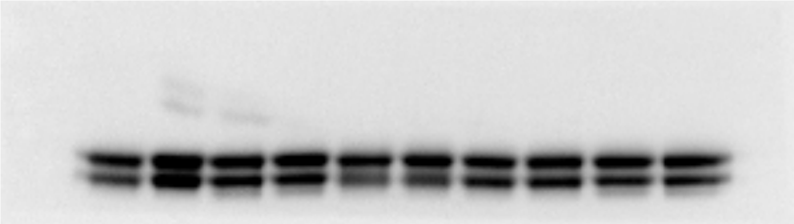

T-BIM

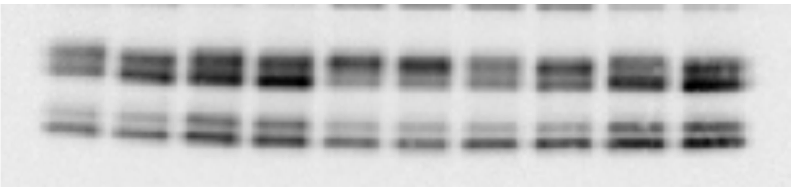

Vinculin

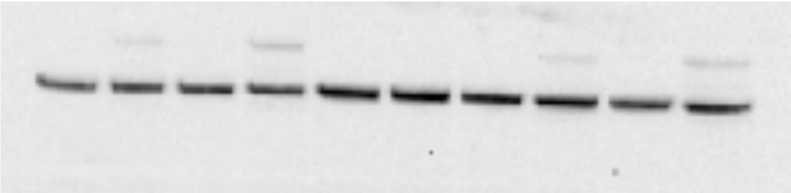

FLAG

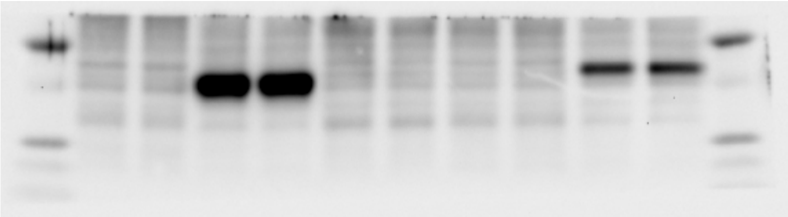

Figure 4:

4C:
p-ERK1/2

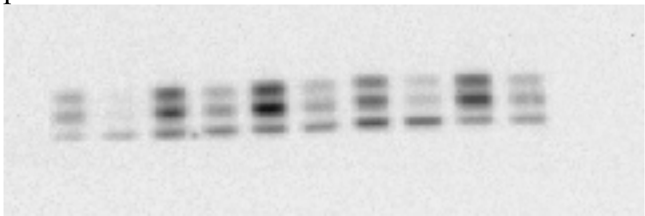

T-ERK1/2

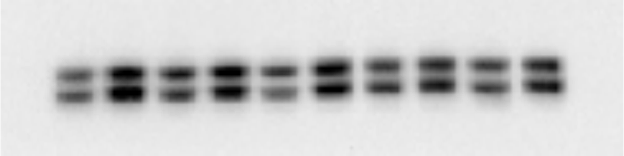

4E:
Vinculin

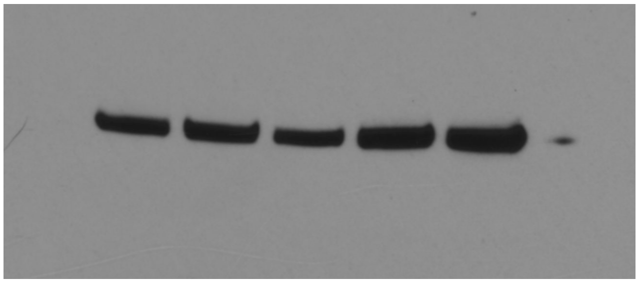

B-actin (top)
FLAG (below)

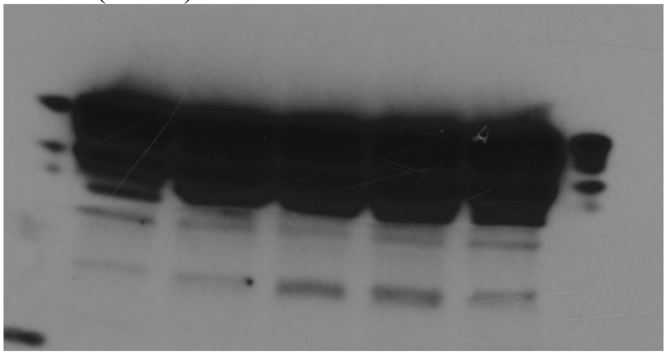

Figure 5:

5B:

Vinculin (top)

B-actin (middle)

KRAS (bottom)

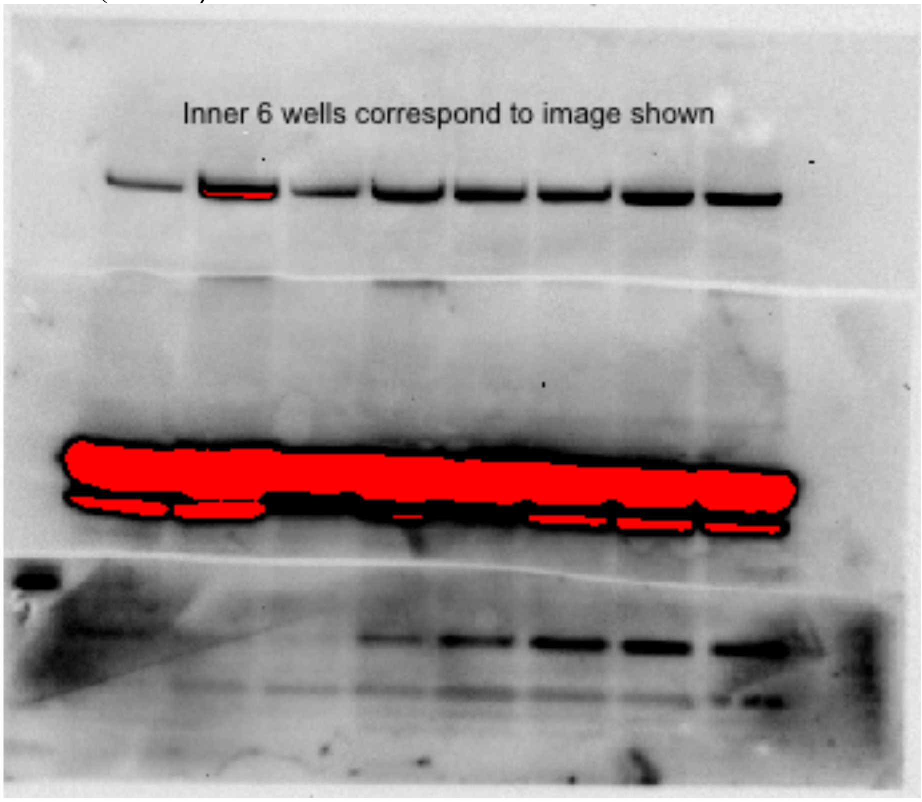

5C:

FLAG

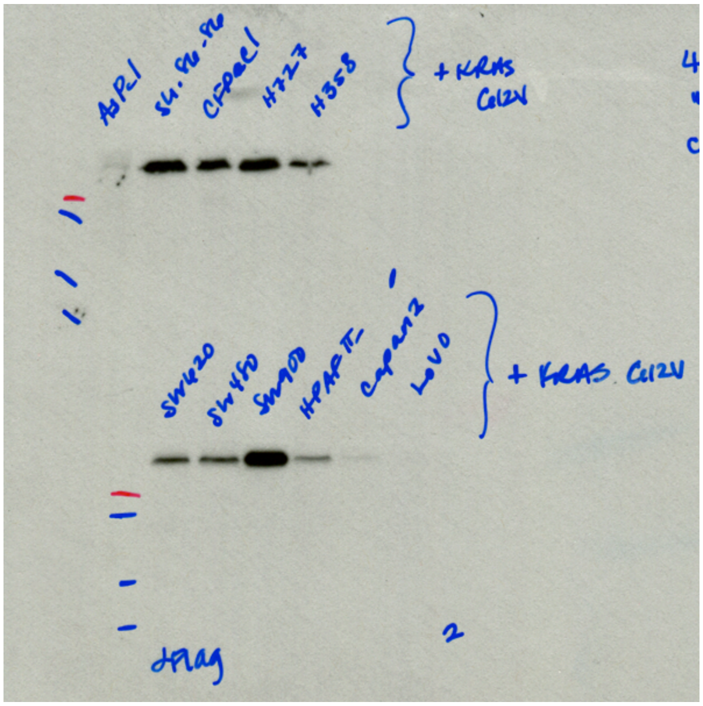

KRAS, B-actin

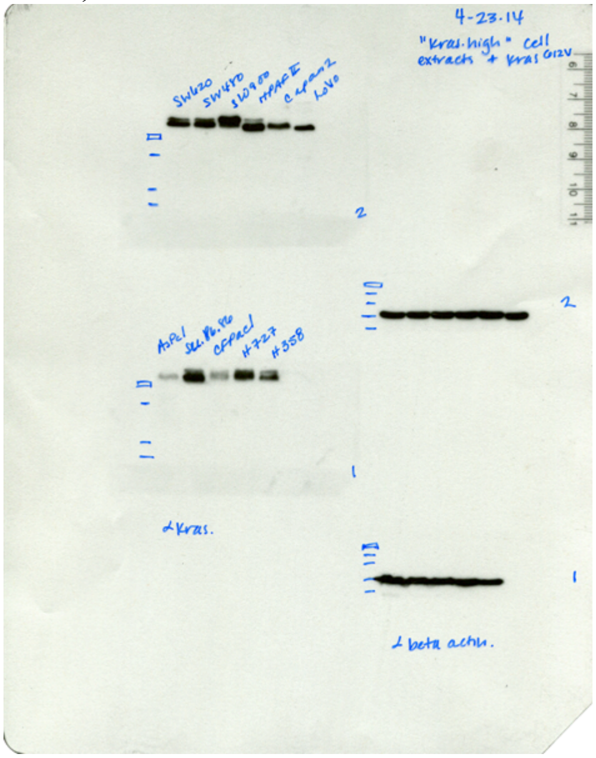

5E:

MYC

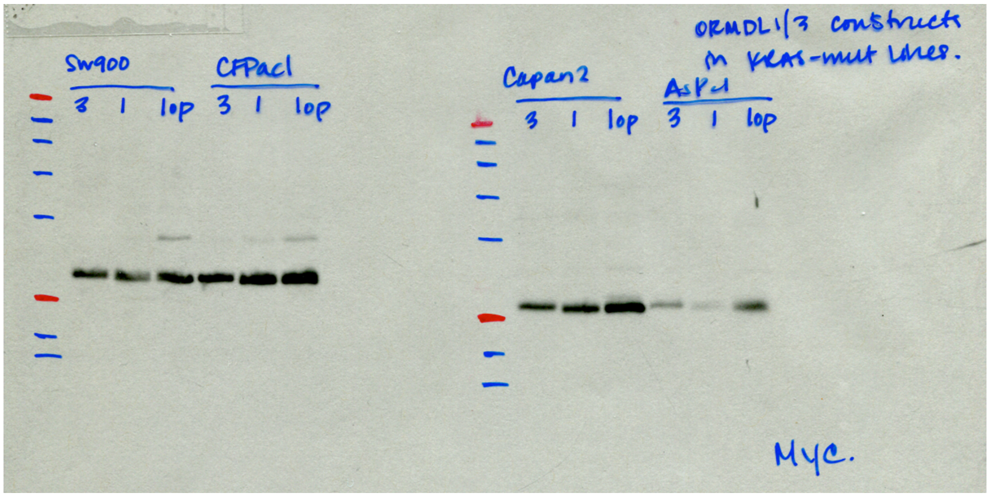

KRAS

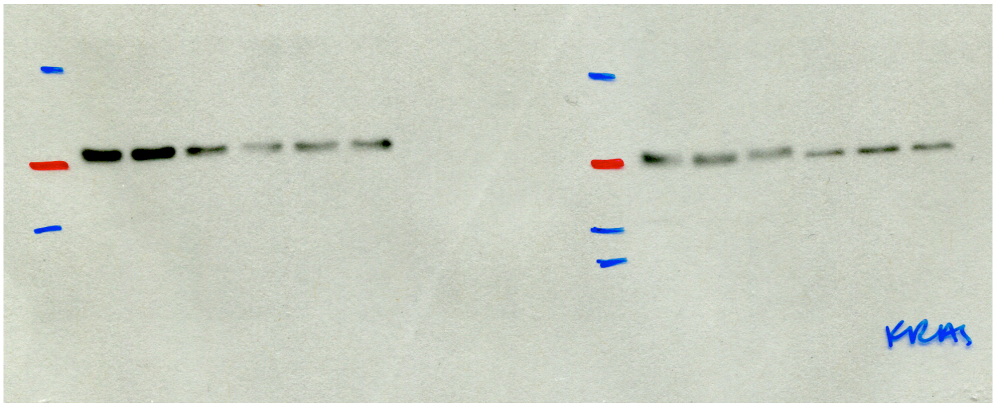

B-actin

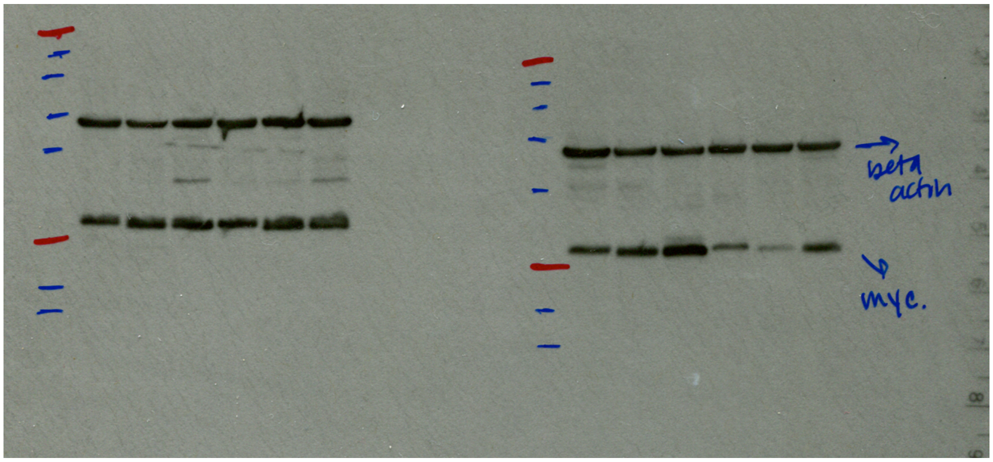

5F:

Puromycin

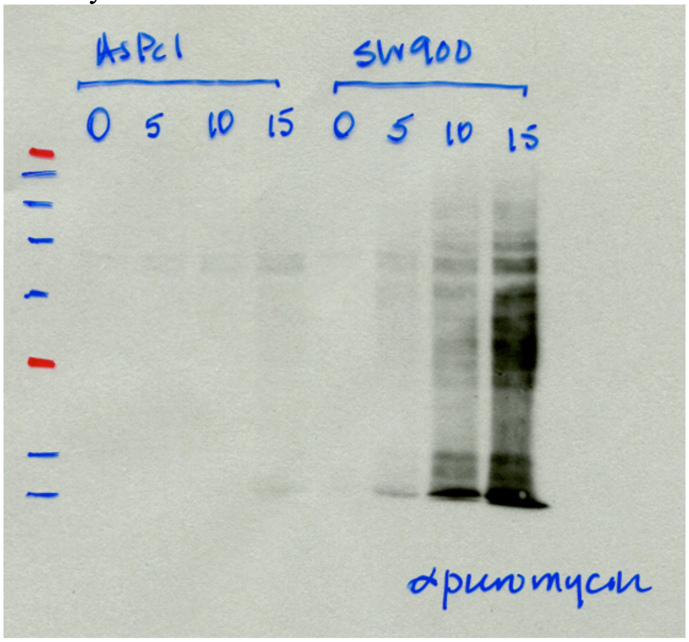

KRAS, B-actin

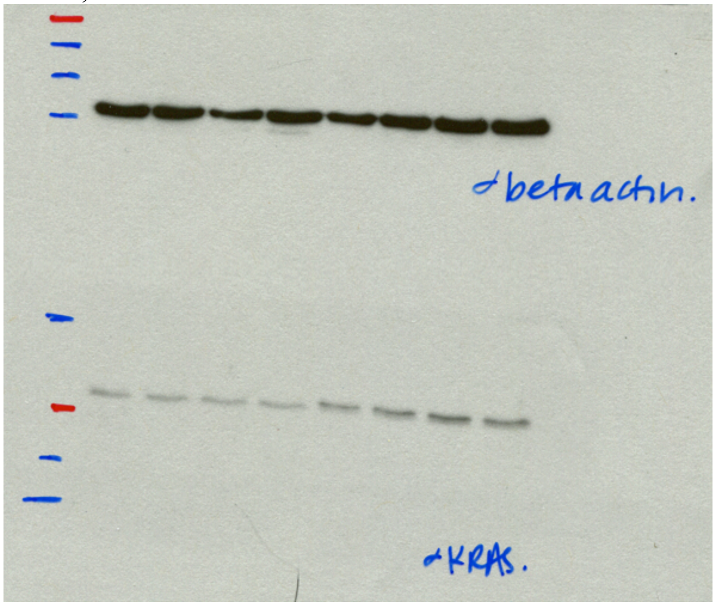

Figure 6A

KRAS

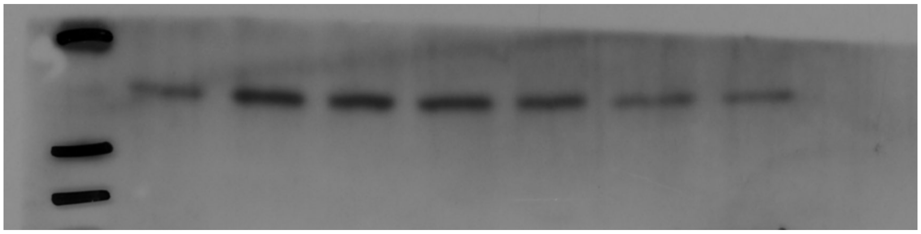

Vinculin

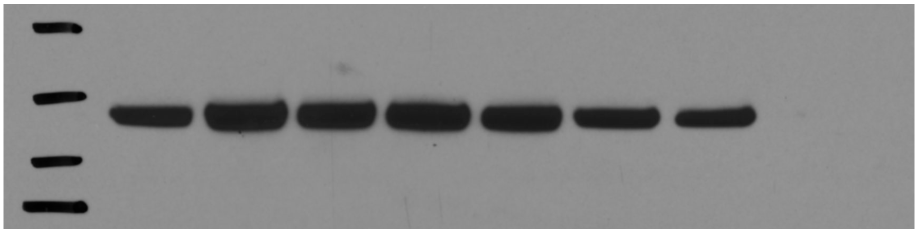

Figure 6E

Vinculin (AZD2014, MLN0128, Rapamycin, 4EGI-1 treatment) (low exposure)

Vinculin (AZD2014, MLN0128, Rapamycin, 4EGI-1 treatment) (high exposure)

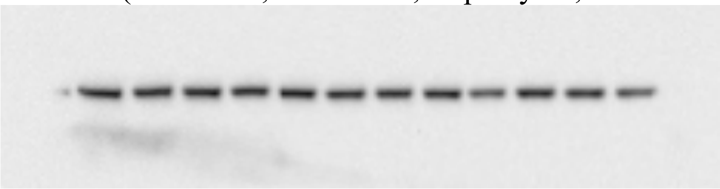

Vinculin (BEZ treatment)

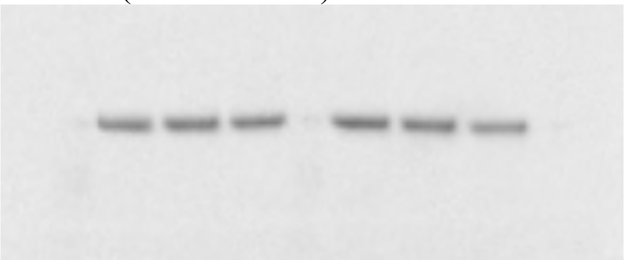

KRAS (BEZ treatment)

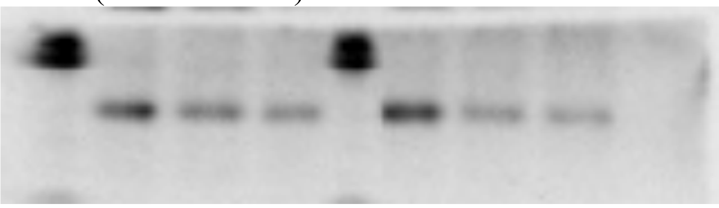

KRAS (P, R1, R2 in the following treatments: AZD2014, MLN0128, Rapamycin, 4EGI-1) (high exposure)

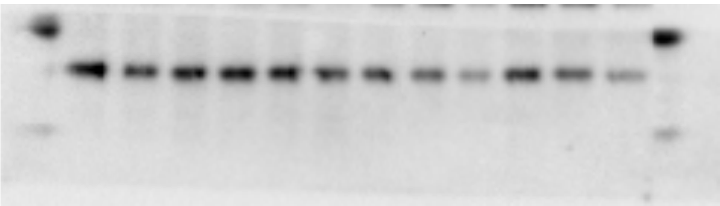

KRAS (P, R1, R2 in the following treatments: AZD2014, MLN0128, Rapamycin, 4EGI-1) (low exposure)

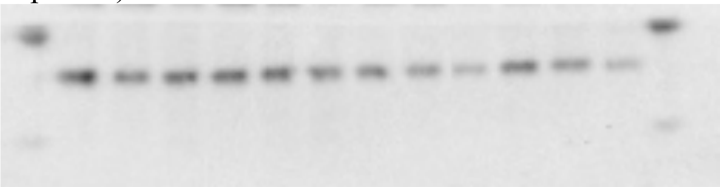

Supplementary Figure 2C:

p-ERK1/2

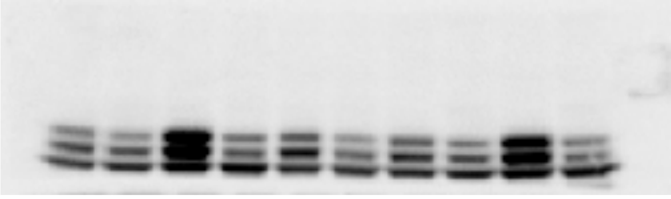

T-ERK1/2

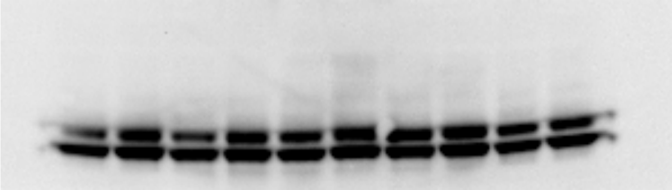

HA

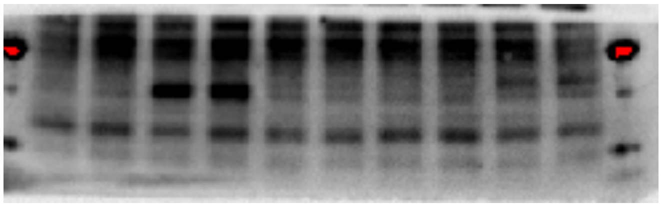

Vinculin

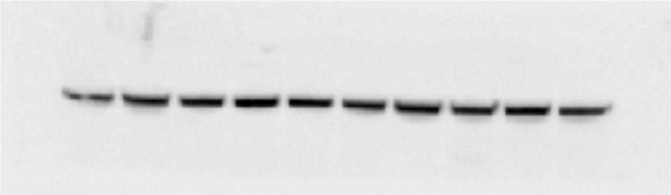

Supplementary Figure 3:

3C:
p-ERK

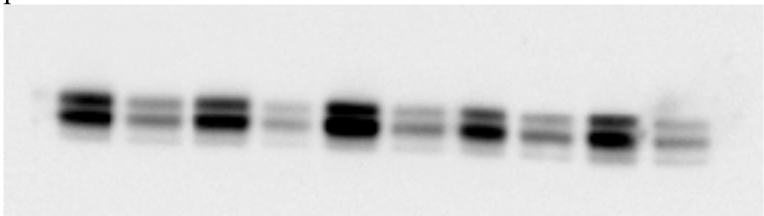

T-ERK

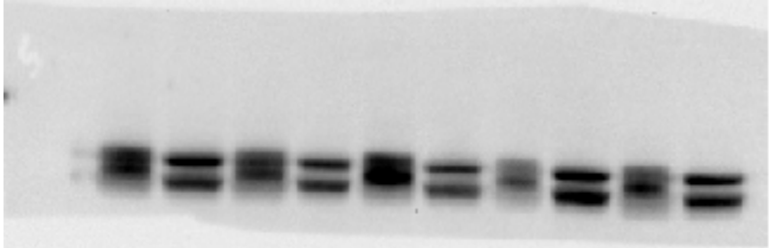

T-BIM

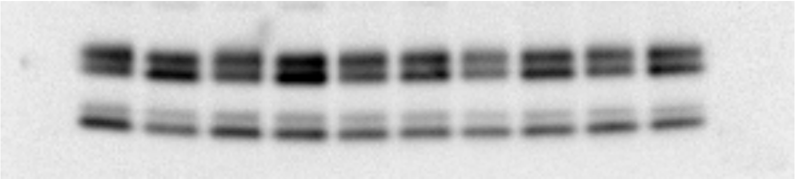

Vinculin

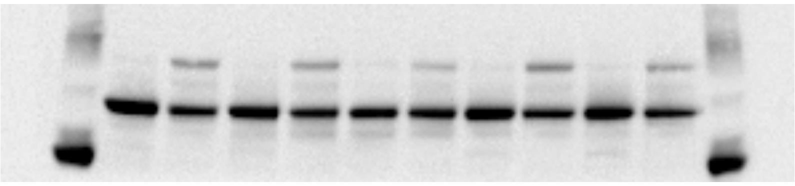

3E:

HA

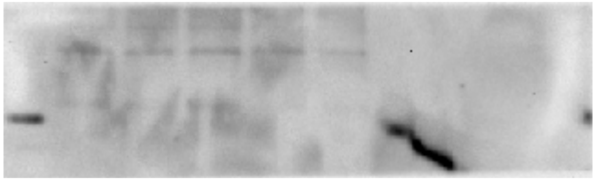

Vinculin

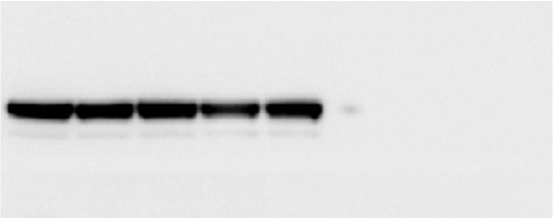

Supplementary Figure 4:

4A:

Puromycin

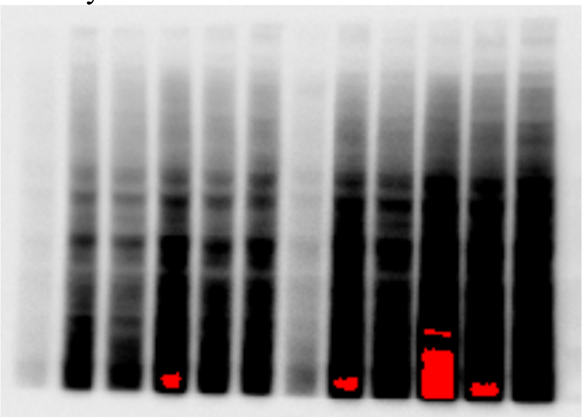

B-actin

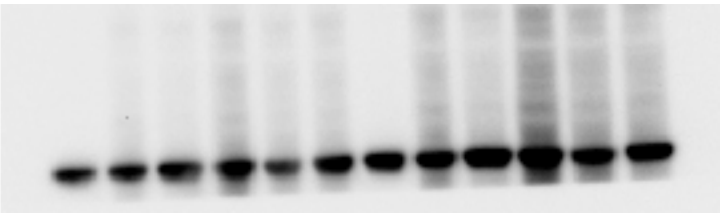

KRAS

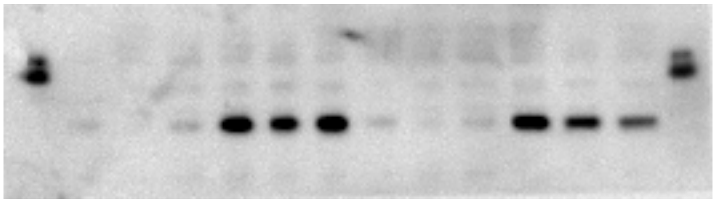

4C:

MYC

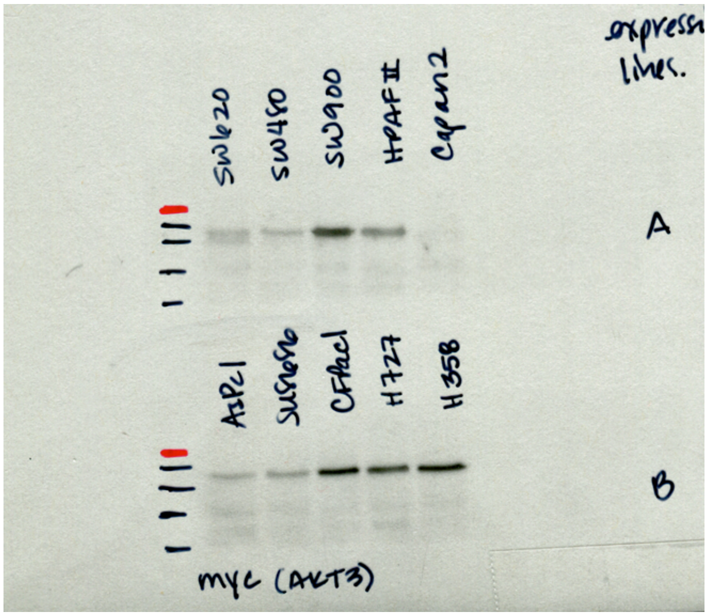

FLAG

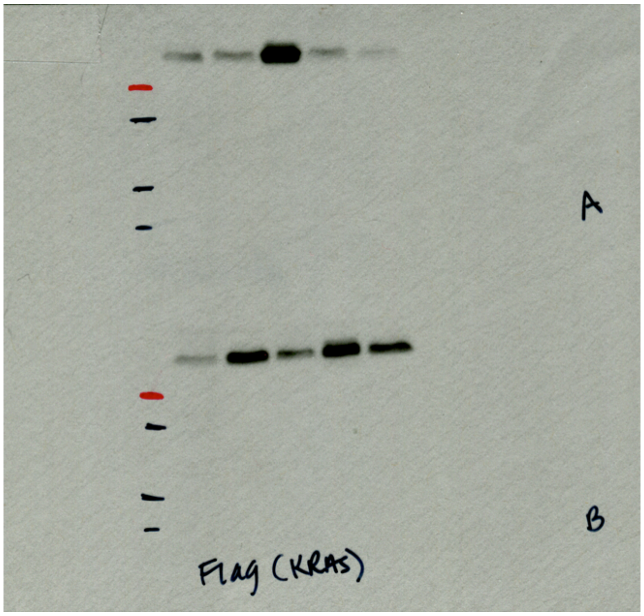

KRAS

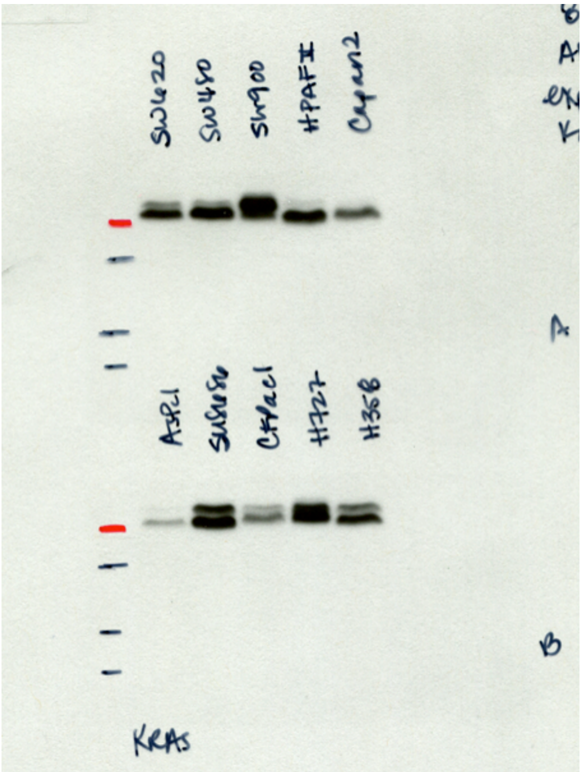
SW620
SW480
SW900
HPAFII
Capan2
A
H727
H358
B
KRAS

B-actin

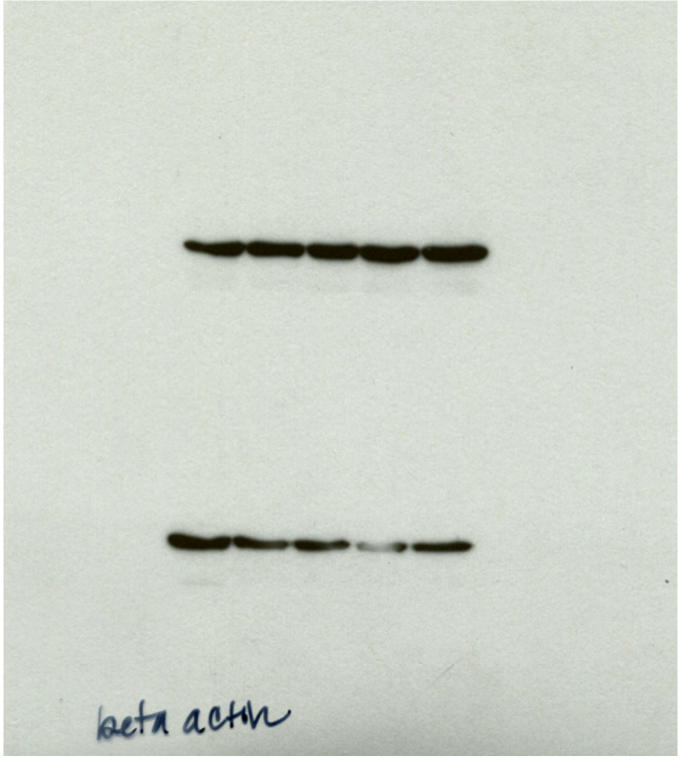
beta actin

4D:

FLAG

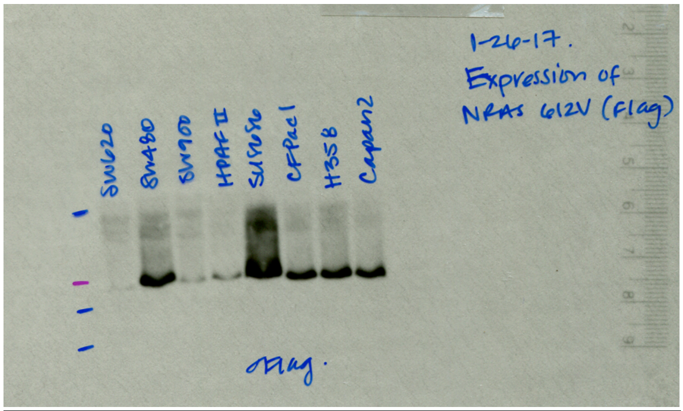

B-actin

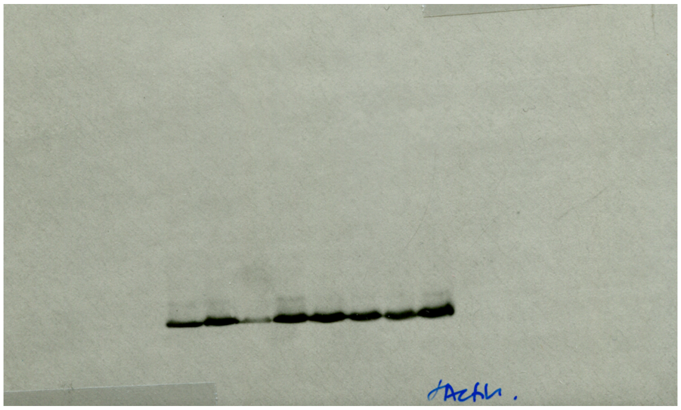

<u>Supplementary Figure 5:</u>

5C:

Puromycin

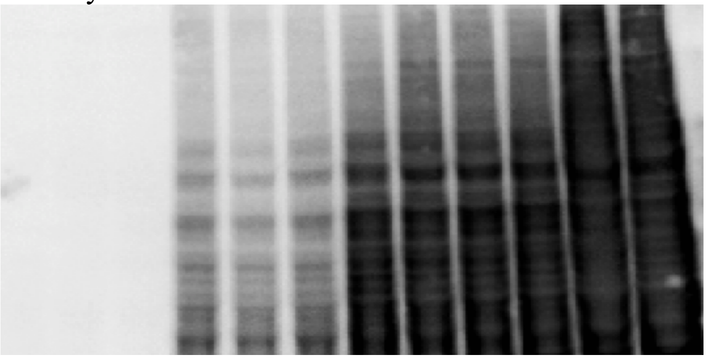

B-actin

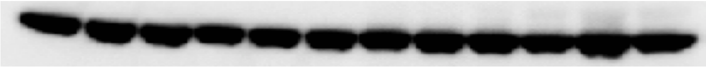

5D:

Vinculin

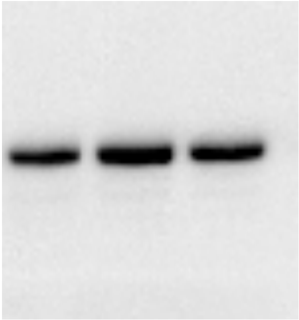

KRAS

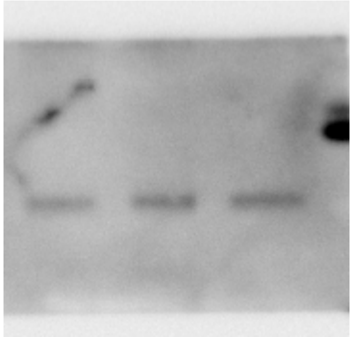

eIF4E

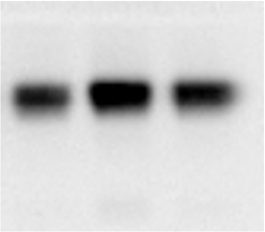

5G:

KRAS

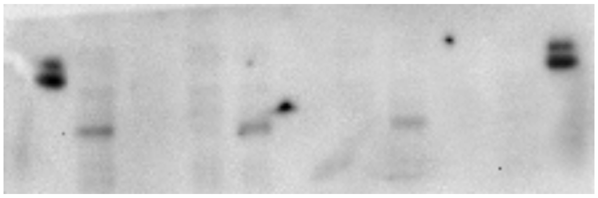

B-actin

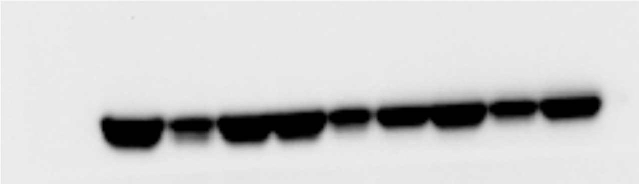

Supplement: Supplementary Information — Supplementary Figures (PDF 20349 kb) [file 41467_2017_BFncomms15617_MOESM307_ESM.pdf]
